# Standard *v*. baby-led complementary feeding: a comparison of food and nutrient intakes in 6–12-month-old infants in the UK

**DOI:** 10.1017/S136898001900082X

**Published:** 2019-05-16

**Authors:** Brigid Alpers, Victoria Blackwell, Miriam E Clegg

**Affiliations:** 1 Oxford Brookes Centre for Nutrition and Health, Oxford Brookes University, Oxford, UK; 2 Department of Food and Nutritional Sciences, University of Reading, Harry Nursten Building, Whiteknights, Reading RG6 6AP, UK

**Keywords:** Baby-led weaning, Infants, Nutrition, Complementary feeding

## Abstract

**Objective:**

To compare food and nutrient intakes of infants aged 6–12 months following a baby-led complementary feeding (BLCF) approach and a standard weaning (SW) approach.

**Design:**

Participants completed an online questionnaire consisting of sociodemographic questions, a 28 d FFQ and a 24 h dietary recall.

**Setting:**

UK.

**Participants:**

Infants (*n* 134) aged 6–12 months (*n* 88, BLCF; *n* 46, SW).

**Results:**

There was no difference between weaning methods for the food groups ‘fruits’, ‘vegetables’, ‘all fish’, ‘meat and fish’, ‘sugary’ or ‘starchy’ foods. The SW group was offered ‘fortified infant cereals’ (*P* < 0·001), ‘salty snacks’ at 6–8 months (*P* = 0·03), ‘dairy and dairy-based desserts’ at 9–12 months (*P* = 0·04) and ‘pre-prepared baby foods’ at all ages (*P* < 0·001) more often than the BLCF group. The SW group was offered ‘oily fish’ at all ages (*P* < 0·001) and 6–8 months (*P* = 0·01) and ‘processed meats’ at all ages (*P* < 0·001), 6–8 months (*P* = 0·003) and 9–12 months (*P <* 0·001) less often than the BLCF group. The BLCF group had significantly greater intakes of Na (*P* = 0·028) and fat from food (*P* = 0·035), and significantly lower intakes of Fe from milk (*P* = 0·012) and free sugar in the 6–8 months subgroup (*P* = 0·03) *v*. the SW group. Fe intake was below the Reference Nutrient Intake (RNI) for both groups and Na was above the RNI in the BLCF group.

**Conclusion:**

Compared with the SW group, the BLCF group was offered foods higher in Na and lower in Fe; however, the foods offered contained less free sugar.

Optimal nutrition in infancy is crucial for growth and development and for establishing good eating habits for long-term health^(^ ^1^ ^)^. At about 6 months of age, infants should be introduced to complementary foods in addition to breast or formula milk, as infant milk alone will not satisfy an infant’s energy and nutrient needs^(^ ^2^ ^)^. Fe and Zn stores in breast milk are almost depleted by 6 months, so complementary foods that provide these micronutrients are of particular importance to the breast-fed infant^(^ ^2^ ^,^ ^3^ ^)^.

The UK Department of Health guidelines on infant feeding recommend breast-feeding exclusively for the first 6 months, after which a variety of complementary foods can be introduced alongside continued breast-feeding (and/or formula milk), but cow’s milk should not be offered as a main drink until after 12 months. Vitamin A, C and D supplements are recommended from 6 months unless the child is formula-fed, and foods should contain no added salt or sugar^(^ ^4^ ^)^.

Traditionally, infants in the UK have been spoon-fed puréed foods and infant cereals as ‘first foods’, but over the past 10–15 years an alternative method of complementary feeding (CF), known commonly as ‘baby-led weaning’, has increased in popularity in countries such as the UK, Canada and New Zealand^(^ ^5^ ^)^. In essence, baby-led complementary feeding (BLCF)* involves finger foods being offered to the infant from the age of 6 months, in addition to continued breast-feeding. The infant is encouraged to join in with family mealtimes and to self-feed as much or as little as his/her appetite allows at each meal^(^ ^6^ ^)^.

It has been suggested that BLCF could be considered a continuation of breast-feeding on demand, which promotes self-regulation of milk volume by the infant^(^ ^7^ ^)^. Proponents of this method assert that because the infant, rather than the adult, is responsible for his/her own feeding, it enables the infant to self-regulate his/her appetite, potentially lowering the risk of obesity later in life^(^ ^6^ ^,^ ^8^ ^)^, while encouraging the development of chewing and fine motor skills^(^ ^9^ ^)^. It has also been suggested that this method introduces infants to a wider variety of foods and textures and may lead to less fussy eating as the child matures^(^ ^6^ ^,^ ^10^ ^)^.

Books and websites on BLCF abound, but due to the lack of research into the nutritional and safety aspects of this method, health professionals are reluctant to recommend BLCF and the main sources of information for parents are BLCF websites and parenting forums^(^ ^10^ ^)^. Because BLCF primarily involves the consumption of finger foods, the main concerns of health professionals are that finger foods could increase the risk of choking and that the energy and Fe intakes of infants might be too low. The advice given by the National Health Service since 2010^(^ ^11^ ^)^ recommends the introduction of soft finger foods from 6 months. Fortified infant cereals such as baby rice are a popular first food for spoon-fed infants but make impractical finger foods. Therefore, another concern is that BLCF infants would lack micronutrients such as Zn and Fe, which fortified cereals contain^(^ ^12^ ^)^. In contrast, parents who are successful in using BLCF report benefits such as it being a less stressful method of feeding than standard weaning^(^ ^12^ ^,^ ^13^ ^)^.

In the UK there have been several large studies investigating the relationship between CF style and behaviour by Brown and Lee^(^ ^10^ ^,^ ^14^ ^,^ ^15^ ^)^, but research into the nutritional adequacy of different feeding methods is scant. One pilot study for a randomized controlled trial has been undertaken in New Zealand to compare nutrient intakes and safety concerns of BLCF and traditionally spoon-fed infants^(^ ^16^ ^)^. That trial concluded that energy intake was similar across both groups, but vitamin A and Se intakes were lower and Na intake higher in the modified BLCF group^(^ ^16^ ^)^. Another small study from New Zealand by Morison *et al*.^(^ ^17^ ^)^ compared nutrient intakes and choking risk of BLCF and traditionally spoon-fed infants, concluding that, although energy intake was similar in both groups, the BLCF group had higher intakes of fat and saturated fat, and lower intakes of Fe, Zn and vitamin B_12_. A further set of studies recently published from the Baby-Led Introduction to Solids (BLISS) trial in New Zealand found that compared with a control group, BLISS infants consumed more Na and fat at 7 months, and less saturated fat at 12 months^(^ ^18^ ^)^. They also found no difference in Zn intake^(^ ^19^ ^)^ but a larger variety of foods offered compared with a control group^(^ ^20^ ^)^. However, this intervention was designed to resolve many of the issues believed to be associated with BLCF and provided guidance and education on the types of foods that could be used to improve the nutritional adequacy of the infant’s diet with particular emphasis on Fe.

Due to the paucity of UK studies comparing food and nutrient intakes of BLCF infants, health professionals and parents have little evidence to recommend this method of CF. The first aim of the present study was to investigate the demographic characteristics of parents in standard weaning (SW) and BLCF groups. The second study aim was to determine whether there are any differences in the foods offered to BLCF and SW infants using data from a validated FFQ. The third aim was to compare the energy and nutrient intakes (protein, carbohydrate, free sugar, fat, saturated fat, Na, Fe, Zn) of infants in each CF group using 24 h dietary recall data.

## Materials and methods

### Study design

The present UK population-based study of infants aged 6–12 months used data collected from parents completing an online questionnaire, consisting of pre-tested demographic questions, questions on feeding style, an FFQ and a 24 h dietary recall.

### Participants

Following obtaining ethical approval from Oxford Brookes University research ethics committee, 320 parents with a child aged 6–12 months were recruited via online parenting websites and posters in nurseries and pre-schools within 10 km of Oxford Brookes University. The poster and information about the study were advertised in a research thread on the websites for Mumsnet and the National Childbirth Trust. Participants were directed to a link to the questionnaire and were invited to complete the questionnaire online or using a paper copy between 31 May and 10 July 2017.

### Exclusion criteria

Parents had to be 18 years of age or over with an infant aged 6–12 months on completion of the questionnaire. They were excluded if their infant was born before 37 weeks’ gestation (premature infants can sometimes be slower to reach milestones such as sitting up or self-feeding^(^ ^21^ ^)^) or had a physical or developmental condition or disability likely to affect their feeding or growth.

### Questionnaire

The questionnaire was formatted using Qualtrics software (Qualtrics^©^ 2017; Provo, UT, USA) and consisted of three main blocks of questions, which took approximately 45 min to complete. The first block consisted of sociodemographic questions about age, ethnicity, academic background and employment status. The questions were devised by the researchers based on similar previous studies^(^ ^22^ ^,^ ^23^ ^)^.

The second block of questions pertained to the infant, including age, sex, weight at birth, current weight, gestation when born, breast-feeding practices and CF methods. The questions regarding CF methods used percentage scales, such as those used by Brown and Lee^(^ ^14^ ^)^: 0, 10, 25, 50, 75, 90 and 100 %. Parents who reported using spoon-feeding for 10 % or less of the time at the infant’s current age were assigned to the BLCF group, whereas those who reported using spoon-feeding more than 10 % of the time were assigned to the SW group.

The third block of questions consisted of an FFQ, validated by previous researchers^(^ ^24^ ^,^ ^25^ ^)^. Permission was granted by Dr Sahota for use in the present study. The FFQ addressed the frequency of consumption of food types and the approximate amount of each food consumed in the past 28 d. A subgroup of participants also completed a 24 h dietary recall, which required participants to recall the foods and drinks their child had consumed in the previous 24 h, excluding foods which were offered, but not eaten.

### Analysis of FFQ

Foods offered per day, week or month were converted into food frequency per day, similar to that calculated by Bingham *et al*.^(^ ^26^ ^)^ in the European Prospective Investigation into Cancer and Nutrition. Foods were assigned to the following groups for analysis of data: all fruits; all vegetables; starchy foods (porridge, breakfast cereal, bread, crackers, breadsticks, chapattis, pita bread, potato, sweet potato, rice, pasta); fortified infant cereals; dairy and dairy-based desserts (cheese, savoury white sauce, yoghurt/fromage frais, ice cream, custard, milk pudding); all fish; oily fish; all meat/fish; processed meats (ham, sausage, bacon, sausage rolls); sugary foods (cakes, biscuits, buns, pastries, sweets); salty snacks (including crisps); pre-prepared baby foods (dried food excluding baby rice, jars, tins, pots or pouches); and sugary drinks (including baby juice, fruit juice, squash and fizzy drinks). Groups were broken down by infant age and CF method, because 6–8-month-old infants will usually be obtaining a higher proportion of energy from milk than foods and are likely to consume less finger foods than 9–12-month-old infants.

### Analysis of 24 h recalls

Fifty participants completed the 24 h dietary recall (BLCF, *n* 29; SW, *n* 21). All diet records were manually entered into Nutritics^®^ dietary analysis software (Nutritics.com 2016, v4.315 Education; Dublin, Ireland). Foods, baby formulas and supplements not listed in Nutritics were defined using supermarket website nutritional information for products per 100 g (Tesco, Asda, Sainsburys and Waitrose). Values for breast milk composition were obtained from McCance and Widdowson’s *The Composition of Foods* ^(^ ^27^ ^)^. To assess the volume of breast milk consumed, the method of Mills and Taylor was applied as described in Lanigan *et al*.^(^ ^28^ ^)^ and Cribb *et al*.^(^ ^29^ ^)^: 135 g breast milk for infants aged 6–7 months and 100 g for those aged 8–12 months were calculated for each feed of at least 10 min duration. Energy and nutrient intakes were obtained and Dietary Reference Values based on the Scientific Advisory Committee on Nutrition/Committee on Medical Aspects of Food Policy reports were calculated in Nutritics. The proportion of food energy from fat, protein and carbohydrate was calculated using 17 kJ/g for protein and carbohydrate and 37·7 kJ/g for fat.

### Statistical analysis

Data were analysed using the statistical software package IBM SPSS Statistics version 23. A *P* value of <0·05 was considered to indicate statistical significance.

#### Demographic data

Chi-squared tests were conducted to test for differences between the SW and BLCF groups where the variables were not a continuous measure (parents’ education, ethnicity, working status, infant sex and breast-feeding status). Independent-samples *t* tests were carried out to examine differences between CF groups on the continuous variables (parents’ age and BMI, number of children, infant gestational age at birth, infant age at the onset of CF, current age, infant birth weight and current weight). Independent-samples *t* tests were used for all parametric data. Mann–Whitney *U* tests were conducted where data were not parametric. Weight-for-age centiles were calculated using the WHO Growth Standards for 0–24 months and significant differences were checked using Mann–Whitney *U* tests.

#### FFQ data

Independent-samples *t* tests for parametric data and Mann–Whitney *U* tests for non-parametric data were used to determine differences between CF groups and the mean number of times infants were offered a food group. The *χ* ^2^ test was used to test for differences in vitamin supplement use between groups.

#### 24 h recall data

Independent-samples *t* tests for parametric data and Mann–Whitney *U* tests for non-parametric data were used to determine differences between CF groups in mean macro- and micronutrient intakes for total intake (food and infant milk), for infant milk only and for food only.

## Results

The questionnaire was attempted by 320 participants (319 online, one paper copy by post). After removing partially completed questionnaires (*n* 173), those in which the infant was born at less than 37 weeks’ gestation (*n* 6) or was older than 12 months (*n* 2) or had allergies or medical conditions which affected feeding (*n* 5), 134 remained. A very limited number of participants indicated the portion size offered at each occasion, so this section of the FFQ had to be discounted. Groups were: SW all (*n* 46), BLCF all (*n* 88); SW 6–8 months (*n* 27), BLCF 6–8 months (*n* 37); SW 9–12 months (*n* 19), BLCF 9–12 months (*n* 51).

Fifty participants gave sufficient detail relating to food, quantity and breast-feeding duration in the 24 h dietary recall: SW all (*n* 21), BLCF all (*n* 29); SW 6–8 months (*n* 13), BLCF 6–8 months (*n* 12); SW 9–12 months (*n* 8), BLCF 9–12 months (*n* 17).

### Demographics

There was no significant difference between CF groups according to parent age, educational level, work status or ethnicity (Table 1). There was no significant difference between CF groups by initial breast-feeding, gestation, age of child at time of filling in questionnaire, infant sex, birth order, birth weight, current weight or centiles for weight and height (Table 2). Infants who followed BLCF commenced weaning significantly later than SW (*P* < 0·001) and significantly more BLCF infants were breast-fed exclusively for 6 months (*P* < 0·001). At the time of the study, in the BLCF group 52 % were consuming breast milk only, 24 % formula only and 24 % were combination feeding (formula and breast milk), whereas in the SW group 43 % were being breast-fed, 43 % formula-fed and 14 % mixed.

**Table 1:** Demographic characteristics for those following standard weaning (SW) and baby-led complementary feeding (BLCF); sample of parents with an infant aged 6–12 months, UK, 31 May–10 July 2017

	SW (*n* 46)		BLCF (*n* 88)		
Parental characteristic	Mean or *n*	sd or %	Mean or *n*	sd or %	*P* value
Age (years), mean and sd †	31·7	4·8	34·0	4·0	0·07
Age group, *n* and %					
<19 years	0	0·0	0	0·0	
20–24 years	0	0·0	6	7·0	
25–29 years	7	15·2	23	26·7	
30–34 years	17	37·0	36	41·9	
≥35 years	22	47·8	21	24·4	
Education, *n* and %					0·70
No formal education	0	0·0	1	1·1	
School GCSE‡	3	6·5	5	5·7	
School A levels§	1	2·2	7	8·0	
College‖	2	4·3	3	3·4	
University	40	87·0	72	81·8	
Ethnicity, *n* and %					0·34
White	43	93·5	84	95·5	
Asian/Asian British	1	2·2	0	0·0	
Black/Black	0	0·0	1	1·1	
African/Black British/Black Caribbean					
Mixed	1	2·2	3	3·4	
Other	1	2·2	0	0·0	
BMI (kg/m^2^), mean and sd	26·1	5·7	26·5	4·8	0·35
BMI category, *n* and %					
<18·5 kg/m^2^	1	2·2	1	1·1	
18·5–24·9 kg/m^2^	20	43·5	36	41·4	
25·0–29·9 kg/m^2^	16	34·8	32	36·8	
30·0–34·9 kg/m^2^	6	13·0	12	13·8	
≥35·0 kg/m^2^	3	6·5	6	6·9	
Number of children, *n* and %					0·364
1	22	47·8	60	68·2	
2	21	45·7	19	21·6	
3	2	4·3	5	5·7	
>3	1	2·2	4	4·5	
Work status, *n* and %					0·25
Full-time	16	34·8	33	37·5	
Part-time	26	56·5	39	44·3	
Not in work	4	8·7	16	18·2	

GCSE, General Certificate of Secondary Education.

† Data from two participants were excluded due to incorrect data entry.

‡ Qualification generally taken by school students in the UK aged 14–16 years.

§ School-leaving qualification in the UK that can be used for university entrance.

‖ Further education generally undertaken between 16 and 19 years that may or may not involve A-level qualifications.

**Table 2:** Infant characteristics for those following standard weaning (SW) and baby-led complementary feeding (BLCF); sample of infants aged 6–12 months, UK, 31 May–10 July 2017

	SW (*n* 46)		BLCF (*n* 88)		
Infant characteristic	Mean or *n*	sd or %	Mean or *n*	sd or %	*P* value
Age (months), mean and sd	8·5	2·0	9·1	1·8	0·07
Age group, *n* and %					
6–8 months	27	58·7	37	42·0	
9–12 months	19	41·3	51	58·0	
Sex, *n* and %					0·47
Male	21	45·7	47	53·4	
Female	25	54·3	42	47·7	
Gestation (weeks), mean and sd	39·5	1·4	40·0	1·4	0·06
Weight-for-age centile at birth, mean and sd	60·6†	26·7	67·0†	27·2	0·14
Weight-for-age centile at current age, mean and sd	58·2‡	28·7	57·5‡	33·0	0·96
Initial breast-feeding, *n* and %	40	87·0	82	93·2	0·23
Exclusively breast-fed for 6 months, *n* and %	15	32·6	56§	64·4	<0·001*
Age of introduction of CF (months), mean and sd	5·5	0·5	5·8‖	0·4	<0·001*

CF, complementary feeding.

* *P* < 0·05 indicates statistical significance.

† Error in data entry: *n* 45 (SW) and *n* 87 (BLCF).

‡ Error in data entry: *n* 45 (SW) and *n* 86 (BLCF).

§ No data for one participant: *n* 87 (BLCF).

‖ Error in data entry for one participant: *n* 87 (BLCF).

### Food frequency

There was no significant difference between weaning methods for food groups of fruits, vegetables, fish, meat and fish, sugary foods or starchy foods (Table 3). The SW group (all ages) was offered significantly more fortified infant cereals (*P* < 0·001), salty snacks at 6–8 months (*P* = 0·03), dairy and dairy-based desserts at 9–12 months (*P* = 0·04) and pre-prepared baby foods at all ages compared with the BLCF group (*P* < 0·001). Conversely, the BLCF group was offered significantly more oily fish at all ages (*P* < 0·001) and 6–8 months (*P* = 0·01), and processed meats at all ages and 9–12 months (*P* = 0·001) and 6–8 months (*P* = 0·003), than the SW group.

**Table 3:** FFQ results: number of times each food type was offered per day over all age groups (total), 6–8 months and 9–12 months, for those following standard weaning (SW) and baby-led complementary feeding (BLCF); sample of infants aged 6–12 months, UK, 31 May–10 July 2017

	Total					6–8 months					9–12 months				
	SW (*n* 21)		BLCF (*n* 29)			SW (*n* 13)		BLCF (*n* 12)			SW (*n* 8)		BLCF (*n* 17)		
Food	Mean	sd	Mean	sd	*P* value	Mean	sd	Mean	sd	*P* value	Mean	sd	Mean	sd	*P* value
Fruits	2·77	0·35	2·37	0·15	0·67	2·35	0·35	1·99	0·20	0·49	3·36	0·66	2·65	0·21	0·37
Vegetables	3·58	0·62	3·49	0·23	0·11	3·22	0·37	3·15	0·56	0·31	4·20	1·28	3·68	0·29	0·33
Fortified infant cereals	0·26	0·58	0·032	0·18	<0·001*	0·19	0·06	<0·001	0·003	<0·001*	0·37	0·11	0·05	0·03	<0·001*
All fish	0·50	0·12	0·50	0·07	0·09	0·36	0·11	0·43	0·12	0·26	0·69	0·20	0·56	0·08	0·46
Oily fish	0·19	0·05	0·23	0·03	<0·001*	0·13	0·05	0·22	0·06	0·01*	0·26	0·09	0·24	0·03	0·12
All meat/fish	1·26	0·20	1·40	0·16	0·33	0·80	0·21	0·91	0·15	0·22	1·91	0·35	1·75	0·35	0·44
Processed meats (ham/sausage/bacon)	0·15	0·05	0·45	0·08	<0·001*	0·10	0·04	0·21	0·04	0·003*	0·22	0·10	0·62	0·12	<0·001*
Sugary foods (cakes/biscuits/snacks)	0·20	0·08	0·22	0·07	0·63	0·10	0·07	0·35	0·03	0·54	0·33	0·15	0·35	0·12	0·77
Salty snacks	0·19	0·05	0·13	0·03	0·31	0·20	0·07	0·05	0·02	0·03*	0·18	0·07	`0·19	0·04	0·96
Starchy foods	3·39	0·40	3·34	0·20	0·28	2·72	0·40	2·42	0·21	0·88	4·34	0·74	4·01	0·28	0·46
Dairy and dairy-based desserts	1·23	0·15	0·98	0·07	0·36	0·89	0·16	0·76	0·08	0·80	1·72	0·25	1·14	0·09	0·04*
Sugary drinks	0·00	0·00	0·02	0·01	0·00	0·00	0·00	0·00	0·00	0·00	0·00	0·00	0·03	0·02	0·16
Pre-prepared baby foods	1·65	0·23	0·49	0·13	<0·001*	1·20	0·23	0·34	0·14	<0·001*	2·29	0·43	0·60	0·20	<0·001*

* *P* < 0·05 indicates statistical significance.

### 24 h dietary recall

There was no significant difference in nutrient intake between weaning methods for energy, carbohydrate, protein, saturated fat or Zn (Table 4). There was a significantly greater intake of free sugar in the 6–8 month SW group (*P* = 0·030), Fe in infant milk in the SW group (*P* = 0·012), fat in food in the BLCF group (*P* = 0·035) and Na in food for the BLCF group (*P* = 0·028). Data were also compared with the Reference Nutrient Intake (RNI) for 7–12-month-old infants (Table 5)^(^ ^30^ ^)^. While mean Zn intake met the RNI for both groups, 50 % of BLCF infants fell below the RNI of 5 mg. Fe intake was lower than the RNI in both groups, but considerably so in the BLCF group.

**Table 4:** 24 h dietary recall results: nutrient intakes over all age groups (total), 6–8 months and 9–12 months, for those following standard weaning (SW) and baby-led complementary feeding (BLCF); sample of infants aged 6–12 months, UK, 31 May–10 July 2017

	Total					6–8 months					9–12 months				
	SW (*n* 46)		BLCF (*n* 88)			SW (*n* 27)		BLCF (*n* 37)			SW (*n* 19)		BLCF (*n* 51)		
Nutrient	Mean	sd	Mean	sd	*P* value	Mean	sd	Mean	sd	*P* value	Mean	sd	Mean	sd	*P* value
Energy (kJ)															
Total	3847·8	1205·9	4143·1	935·0	0·33	2368·3	924·7	237·7	899·0	0·99	1479·5	9354·6	1770·4	850·3	0·26
Infant milk	2368·3	924·7	2372·7	899·0	0·99	2649·8	836·4	2654·8	960·3	0·99	1910·8	925·8	2173·5	823·7	0·48
Food	1479·5	935·6	1770·4	850·3	0·26	1132·0	872·8	1127·9	498·8	0·99	2044·3	778·7	2223·9	752·4	0·59
Carbohydrate (g)															
Total	111·2	32·6	112·5	30·2	0·88	108·8	31·4	96·5	22·4	0·40	115·2	36·1	121·9	32·0	0·66
Infant milk	62·1	20·8	60·2	23·1	0·77	68·7	18·1	60·6	23·6	0·30	51·3	21·4	55·5	22·0	0·66
Food	49·1	30·3	52·3	30·4	0·72	40·1	30·3	32·4	16·9	0·45	63·9	25·4	66·3	30·3	0·85
Protein (g)															
Total	25·9	9·9	27·5	8·5	0·47	23·5	9·3	22·6	5·5	0·77	29·8	10·2	31·1	8·8	0·75
Infant milk	11·1	3·8	10·8	4·0	0·79	12·3	3·3	12·0	4·3	0·84	9·0	4·0	9·9	3·7	0·61
Food	14·8	9·5	16·8	9·3	0·46	11·2	8·9	10·6	6·3	0·86	20·7	7·5	21·2	8·6	0·91
Fat (g)															
Total	41·0	16·9	47·7	13·7	0·12	41·7	14·5	46·1	11·9	0·54	39·7	21·3	48·8	15·1	0·23
Infant milk	30·7	15·0	31·8	12·6	0·64	34·9	14·1	35·8	13·7	0·94	24·0	14·5	29·0	11·3	0·19
Food	10·2	8·4	15·9	9·8	0·04*	6·8	6·2	10·2	5·1	0·12	15·7	9·0	19·8	10·5	0·55
Saturated fat (g)															
Total	16·7	7·6	19·8	6·9	0·13	17·5	6·7	18·9	5·5	0·57	15·2	9·3	20·5	7·9	0·09
Infant milk	12·9	6·5	13·7	5·5	0·56	14·6	6·2	15·6	5·9	0·85	10·2	6·6	12·4	5·0	0·22
Food	3·7	3·4	6·1	5·3	0·08	2·9	3·4	3·4	2·2	0·41	5·1	3·1	8·0	6·1	0·24
Free sugar (g)															
Total	5·2	7·5	3·6	5·1	0·58	6·5	9·0	1·0	2·1	0·03*	3·0	3·4	5·5	5·8	0·29
Fe (mg)															
Total	6·2	4·9	4·8	2·6	0·25	6·3	5·8	4·2	3·0	0·57	6·0	3·2	5·3	2·2	0·51
Infant milk	2·4	1·7	1·6	1·9	0·01*	2·1	1·5	1·8	2·1	0·12	2·9	2·1	1·5	1·9	0·08
Food	3·8	4·5	3·2	2·2	0·55	4·2	5·6	2·5	1·9	0·85	3·1	2·0	3·7	2·3	0·55
Zn (mg)															
Total	5·8	2·9	5·2	1·9	0·40	6·0	3·3	5·0	2·1	0·47	5·4	2·3	5·3	1·8	0·95
Infant milk	5·4	2·3	5·3	1·8	0·05	3·6	0·5	3·3	1·6	0·66	2·9	0·9	2·8	1·5	0·32
Food	2·5	2·7	2·2	1·4	0·52	2·5	3·3	1·7	1·4	0·94	2·5	1·8	2·6	1·4	0·92
Na (mg)															
Total	375·5	219·4	529·1	224·8	0·01*	315·3	161·9	391·2	117·1	0·10	473·4	273·7	626·5	233·9	0·32
Infant milk	134·6	42·3	129·9	55·6	0·76	149·5	36·2	145·8	54·5	0·84	110·3	50·1	118·8	55·2	0·71
Food	240·9	218·0	399·1	237·0	0·03*	165·7	166·7	245·4	127·2	0·23	363·0	246·3	507·6	238·7	0·18

* *P* < 0·05 indicates statistical significance.

**Table 5:** Comparison of total nutrient intakes from 24 h dietary recalls with recommendations for those following standard weaning (SW) and baby-led complementary feeding (BLCF); sample of infants aged 6–12 months, UK, 31 May–10 July 2017

	6–12 months				
	SW		BLCF		
Nutrient	Mean	sd	Mean	sd	RNI^(^ ^30^ ^)^
Energy (kJ)	3847·8	1205·9	4143·1	935·0	2853†
Protein (g)	25·9	9·9	27·5	8·6	14·3‡
Fe (mg)	6·21	4·90	4·84	2·60	7·8
Zn (mg)	5·8	2·2	5·2	1·9	5·0
Na (mg)	375·5	219·4	529·1	228·8	400
Ca (mg)	588·9	209·1	579·9	211·8	525
Mg (mg)	80·9	29·4	90·7	27·1	75
Vitamin A (μg)	787·0	327·5	687·7	192·1	350
Vitamin B_12_ (μg)	1·43	0·89	1·47	1·03	0·4
Vitamin C (mg)	86·9	36·9	80·6	30·3	25

RNI, Reference Nutrient Intake.

† Energy given as Estimated Average Requirement.

‡ Average of male and female requirements, mixed feeding, 7–12 months^(^ ^30^ ^)^.

### Proportion of food energy from macronutrients

The BLCF group obtained a greater percentage of energy from fat (34 %) than the SW group (26 %) and less from carbohydrate (50 %) than the SW group (57 %). The proportion of energy from protein was similar in both groups (BLCF, 16 %; SW, 17 %). Free sugars in the SW group accounted for 9 % of energy intake, considerably higher than the 1 % for the BLCF group.

### Supplements

Seventy per cent of BLCF infants were given multivitamin or vitamin D supplements, compared with 48 % of SW infants, which showed a trend towards statistical significance (*P* = 0·05).

### Salt

The proportion of parents who reported never adding salt during the preparation of infant food was similar for the SW group (84 %) and the BLCF group (85 %).

## Discussion

Our findings indicate some differences in food and nutrient intakes between BLCF and SW infants. This section first considers the demographic data of the population and their feeding styles, then any differences in macronutrients and micronutrients and food sources between the groups, before examining the limitations of the study.

The questionnaire tended to attract parents with a preference towards BLCF, with 66 % of participants following a BLCF approach, despite CF methods not being mentioned on the recruitment poster. While the demographic characteristics of the present study groups (SW, BLCF) were well matched for age, education, work status, ethnicity and sex of infant, they are not representative of the UK population as a whole. Comparing Office for National Statistics^(^ ^31^ ^)^ figures from the 2011 census with results from our study, 94·8 % of the participants were white, compared with the national average of 86 %, and 83·7 % had held a university degree compared with 27 % nationally. There is evidence that parents who choose BLCF in the UK have more years of education^(^ ^14^ ^)^.

In our study, and in previous research^(10,14,17,32)^, BLCF was associated with a longer duration of breast-feeding and a later introduction of complementary foods, both of which are considered beneficial to infant health^(^ ^14^ ^)^. Sixty-four per cent of BLCF infants were breast-fed exclusively for the first 6 months, compared with 32 % of SW infants and only 1 % in the 2010 Infant Feeding Survey^(^ ^33^ ^)^. BLCF infants were first introduced to complementary foods at an average age of 5·8 months, which was later than the SW group (5·5 months) but in line with the recommended age of about 6 months. However, in 2010 in the UK, 75 % of infants had been introduced to CF by the age of 5 months^(^ ^33^ ^)^. Seventy per cent of BLCF parents reported giving their infants vitamin supplements as recommended for all breast-fed infants compared with only 48 % of SW parents, although some parents noted that they did not remember to do this every day.

The study indicated that there were no differences between BLCF and SW in terms of energy intake, but the proportions of energy from macronutrients in food and the types of foods offered were different. BLCF infants were offered significantly more fat in food than SW infants, which agrees with the findings of Morison *et al*.^(^ ^17^ ^)^. From the age of 2 years onwards, fat as a percentage of energy intake should be no more than 35 %^(^ ^30^ ^)^. Both BLCF and SW infants met this guidance: BLCF infants (all ages) derived 34 % of food energy from fat, compared with 26 % for SW infants. Although these are just estimates of dietary intakes, 26 % of energy from fat in the diet is relatively low as studies have shown that infants on a low-fat diet (25 % or less energy from fat) commonly fail to thrive^(^ ^34^ ^,^ ^35^ ^)^.

The Scientific Advisory Committee on Nutrition’s 2015 report^(^ ^36^ ^)^ states that from the age of 2 years, free sugars should amount to no more than 5 % of total energy (there is no guidance for children under 2 years). Free sugars accounted for only 1 % of total energy in the BLCF group; however, this was 9 % in the SW group. Commercially prepared baby foods were offered 11·6 times weekly for SW infants compared with only 3·4 times weekly for BLCF infants, potentially providing less free sugar. Crawley and Westland^(^ ^37^ ^)^ criticized manufacturers of commercially prepared baby foods in the UK for adding fruit to provide sweet flavours to vegetable-based purées, resulting in a high concentration of sugar. The authors also commented that these foods are unlikely to replicate the taste and texture of homemade food and may have a negative influence on dental health if sucked directly from a baby food pouch. Studies by Coulthard *et al*.^(^ ^38^ ^,^ ^39^ ^)^ showed that introducing homemade foods with ‘lumps’ and varied textures before 9 months increased both the range of foods and the quantity of fruit and vegetables that a child will consume at 7 years compared with infants fed solely on puréed foods. In contrast, Smithers *et al*.^(^ ^40^ ^)^ used data from the Avon Longitudinal Study of Parents and Children to show that 6–8-month-old infants who consumed more ready-prepared baby foods had lower Na and higher Fe intakes than infants consuming breast milk and homemade foods.

In the present study, BLCF infants consumed a mean of 529·11 mg Na/d (or 1·3 g salt/d), which is one-third above the daily recommended maximum of 400 mg. Results from the FFQ showed that BLCF infants were offered more processed meats, a known source of Na and nitrate in the diet^(^ ^41^ ^)^, than SW infants. In the short term, Na intake above 400 mg/d in infants may cause harm to developing kidneys, and in the long term a preference for salty foods may result in problems such as high blood pressure in adulthood^(^ ^42^ ^)^. Na intake ranged from 154 to 1102 mg/d in the BLCF group, with two infants consuming almost three times the recommended intake of Na in 24 h (1102 mg and 1082 mg). In this case, the majority of the Na was contained in baked beans, ham, crumpets and cheese. Added Na is rarely present in commercially puréed baby foods, which represented a greater proportion of dietary intake in the SW group than the BLCF group, but could be present in family food unmodified for BLCF infants. Cribb *et al*.^(^ ^29^ ^)^ calculated intake of Na and Fe from 3 d dietary records of family foods offered to 8-month-old infants (*n* 1178) and 70 % consumed more than the daily maximum of 400 mg. However, 85 % of BLCF parents in the current study reported that they never added salt to food, although the BLCF group was offered processed meats on average just over three times per week. The mean Na intake for SW, 375 mg/d, was in line with the RNI.

Fe is required for the development of erythrocytes, immune function and cognitive development^(^ ^43^ ^)^. Fe-deficiency anaemia, caused by insufficient dietary Fe, can lead to delays in the development of cognitive function which can be irreversible^(^ ^44^ ^)^. The UK has no screening policy for Fe deficiency, which makes it difficult to estimate the prevalence in the population^(^ ^45^ ^)^, but in the 2011 Diet and Nutrition Survey of Infants and Young Children, Fe intake was 10–14 % below the Lower Reference Nutrient Intake^(^ ^46^ ^)^. In our study, Fe intake was below the RNI for both groups: the SW group was 20 % below the RNI and the BLCF group was 38 % below the RNI. The lower Fe intake in the infant milk portion of the BLCF group could be explained in part by a greater consumption of breast milk, which has a lower concentration of Fe than formula milk (approximately 0·07 mg/100 ml compared with 0·80 mg/100 ml in formula milk^(^ ^47^ ^)^) and lower intake of commercially prepared baby foods and fortified infant cereals. This suggests that BLCF infants may need foods with a greater Fe content, especially if breast milk is still a large part of overall energy intake. The FFQ showed significantly more fortified infant cereal (baby rice) offered to the SW group, which is a good source of Fe, but is difficult for BLCF infants to consume when self-feeding. Compared with the RNI for 7–12-month-old infants, average Zn intake met the RNI for both groups, but 50 % of BLCF infants fell below the RNI of 5 mg. Red meat, such as beef and lamb, is a good source of Fe and Zn, but it can prove difficult to chew and parents may worry about infants choking if it is in finger-food form.

Data for the present study were self-reported and could be open subject to error (e.g. people misreporting or estimating body weight). The participants for the study were also self-selected and the choice of weaning style was also selected by the participants. While Internet recruitment is efficient, it may be biased towards participants who have a higher level of education^(^ ^48^ ^)^. Although 320 surveys were attempted, only 134 were fully completed. The length of the questionnaire was a limitation and many participants completed the demographic questions but did not progress further to the FFQ. The fifty participants who completed the entire survey including the 24 h dietary recall were those who were more motivated to do so, and this may have biased the results. The FFQ has not been validated for online use and as such the decision was taken to focus solely on the types of foods consumed from these data. The nutrients contained in breast milk are very difficult to standardize since the composition of breast milk changes between each feed and the fat content of milk varies as the breast is emptied of milk^(^ ^47^ ^,^ ^49^ ^)^. Assessing the accuracy of duration and volume of breast milk is difficult. It is likely that some participants overestimated the duration of feeds or the time the infant was actively sucking. However, energy from milk and food was similar for both BLCF and SW infants, which suggests the method was consistent with volumes calculated for formula milk. The 24 h recall data were dependent on participants recording the quantity of food actually ingested, which is problematic with infants, so the quantities stated can only be estimates. The habitual intake of foods consumed is difficult to estimate in a 24 h food recall; a longer (2 d) weighed food diary would be a more accurate indicator of quantity ingested, but would require many more resources than were available in the present study. The questionnaire was undertaken at any time when the child was 6–12 months old and it is known that babies will transition from being spoon-fed (SW) to self-feeding (BLCF) during this time^(^ ^50^ ^)^. Future studies should assess food intake at the point of weaning.

As an area in which research is limited, and the first study of this type in the UK, the present study supplements the published evidence currently available on nutrient intakes of infants following BLCF and SW approaches to CF. The survey was comprehensive, which meant a broad range of data could be collected. The sample size was larger than for similar studies, such as that of Morison *et al*.^(^ ^17^ ^)^, which gives the study more statistical power. Finally, all demographic data were consistent between groups for parents, and age, sex and weight of infants were consistent between groups.

## Conclusion

Doctors, midwives and health visitors are reliant on evidence-based research to inform their advice to parents. The present study adds to the small pool of knowledge relating to food and nutrient intakes and CF methods. The study suggests that BLCF can have both positive and negative implications for the diets of infants. Parents need to be made more aware of the types of foods they should or should not be offering their infant to ensure that Na intake is not too high and that Fe intake is sufficient. In the current study the BLCF group was less likely to be offered commercially prepared baby foods and less free sugar than the SW group. Parents using BLCF should be informed of the benefits and limitations and given advice to ensure optimal nutritional intake during this important time such as has been achieved during the BLISS studies^(^ ^16^ ^)^.

## Footnotes

* The term baby-led complementary feeding (BLCF) is used throughout the present paper as babies are still being breast-fed, are not yet weaned, but they have been introduced to complementary feeding.
